# Supplementary material for: A Phenotype-Structured PDE Framework for Investigating the Role of Hypoxic Memory on Tumor Invasion under Cyclic Hypoxia
Source: Bull Math Biol. 2026 Jan 14;88(2):23. doi: 10.1007/s11538-025-01591-2 (PMC12804273; doi:10.1007/s11538-025-01591-2)
Supplement: Supplementary file 1 — (pdf 3313 KB) [file 11538_2025_1591_MOESM1_ESM.pdf]

## Supplementary Information

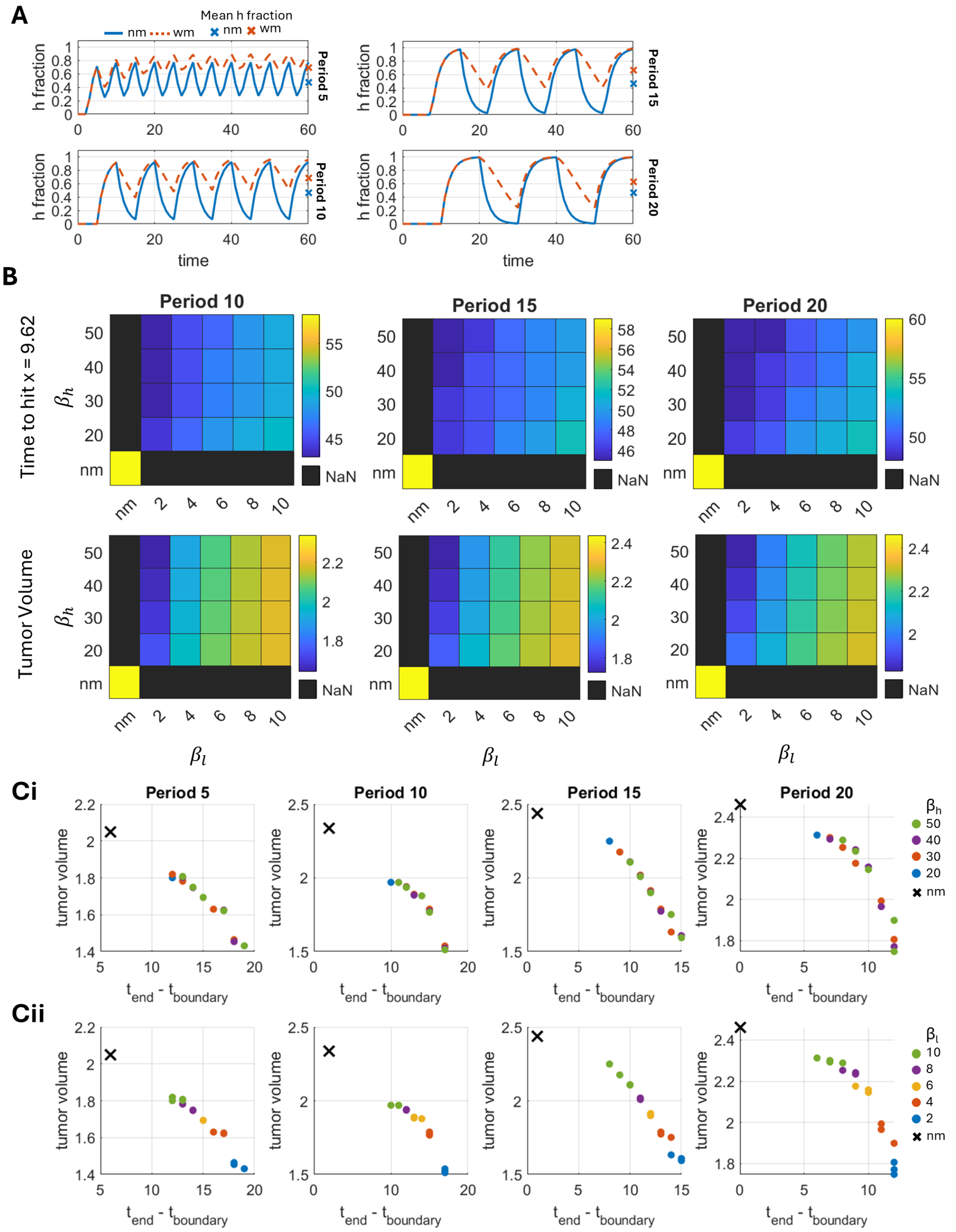


**Fig. S1**: A) Temporal changes in the hypoxic cell fraction in the tumor bulk for increasing time period. ‘nm’ represent no hypoxic memory case and ‘wm’ denotes hypoxic memory with $\beta_1 = 20$ and $\beta_1 = 2$. Mean h fraction is calculated by averaging h fraction over the simulation period. B) Changes in the tumor volume and time to hit at a physical distance of x = 9.62 (minimum invasion length obtained for $\beta_h$=20 and $\beta_l$=10) for different combinations of memory induction, erasure timescales and environment period. C) Scatter plot of the data presented in Figure 2D and panel B above. In panels Ci and Cii points are labeled with values of parameters $\beta_h$ and $\beta_l$, respectively. Cross mark ‘x’ represent the tumor volume and hitting time for without hypoxic memory case. $t_{end}$ is the total simulation of 60 units and $t_{boundary}$ is the time to hit boundary (x = 9.62).

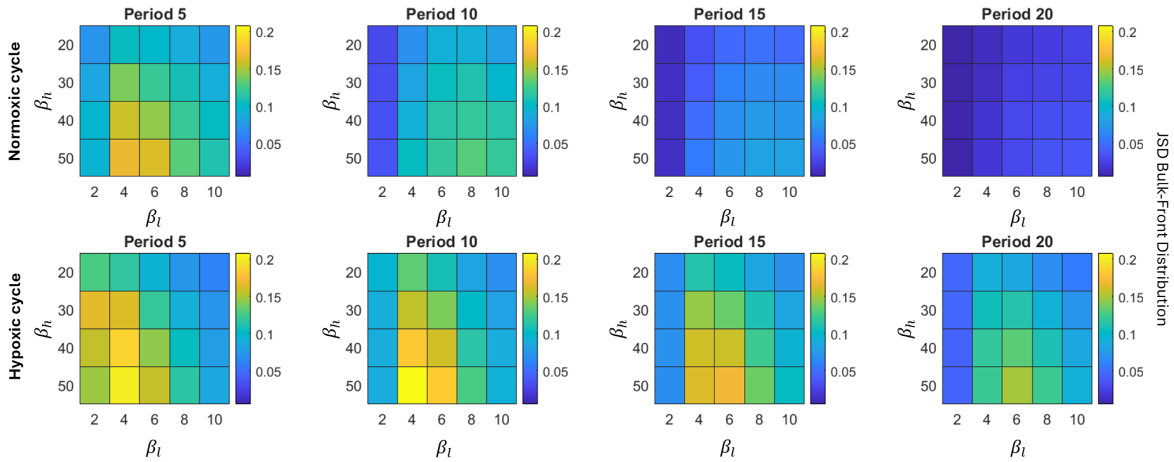


**Fig. S2**: Using Jensen-Shannon Divergence to quantify the differences in phenotypic distributions of hypoxic cells between the tumor front and bulk cells at end of the normoxia and hypoxia environmental cycles. The hypoxia period bias is set to 0.5.

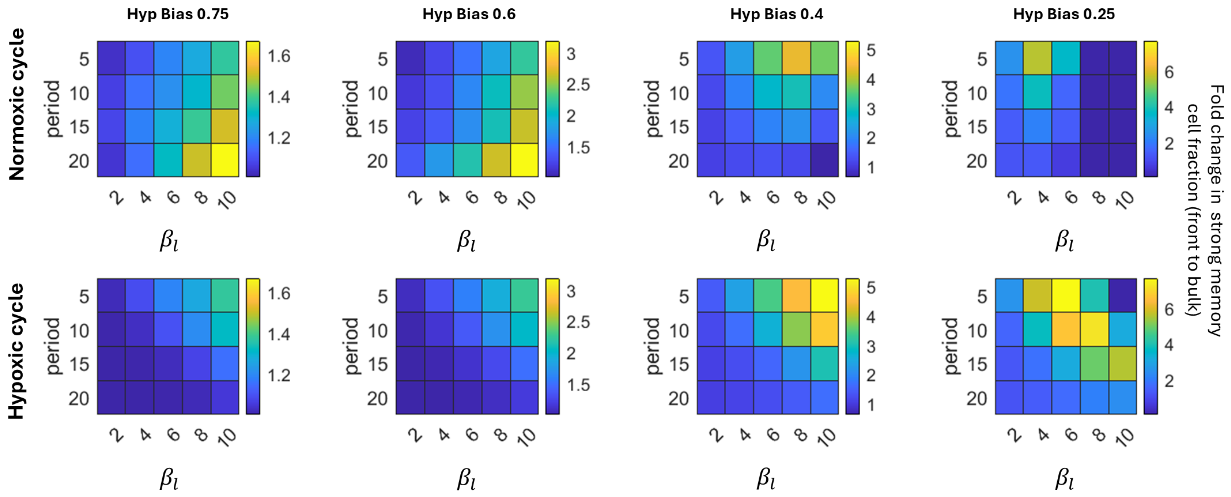


**Fig. S3**: Quantification of ratio of strong memory cell fraction in the front to bulk regions at the end of normoxia and hypoxia cycles of different environmental bias. Here, the strong memory fraction is calculated by summing normalized cell density with $\mu_{hn} < 0.25$. Results presented are with $\beta_h = 20$.

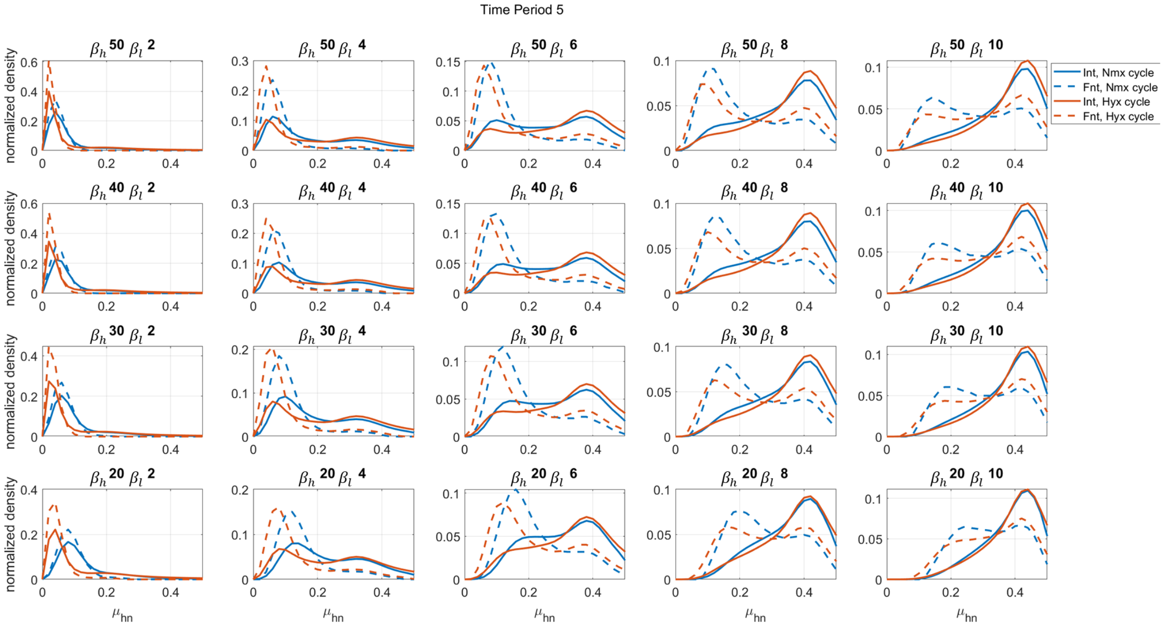


**Fig. S4**: Normalised phenotypic ($\mu_{hn}$) distribution of cells in the bulk (total population) and front (defined by width of 0.5 unit distance from the right boundary) analysed at the end of the normoxic and hypoxic cycles of the last period of the simulation. Here the environment period is 5.

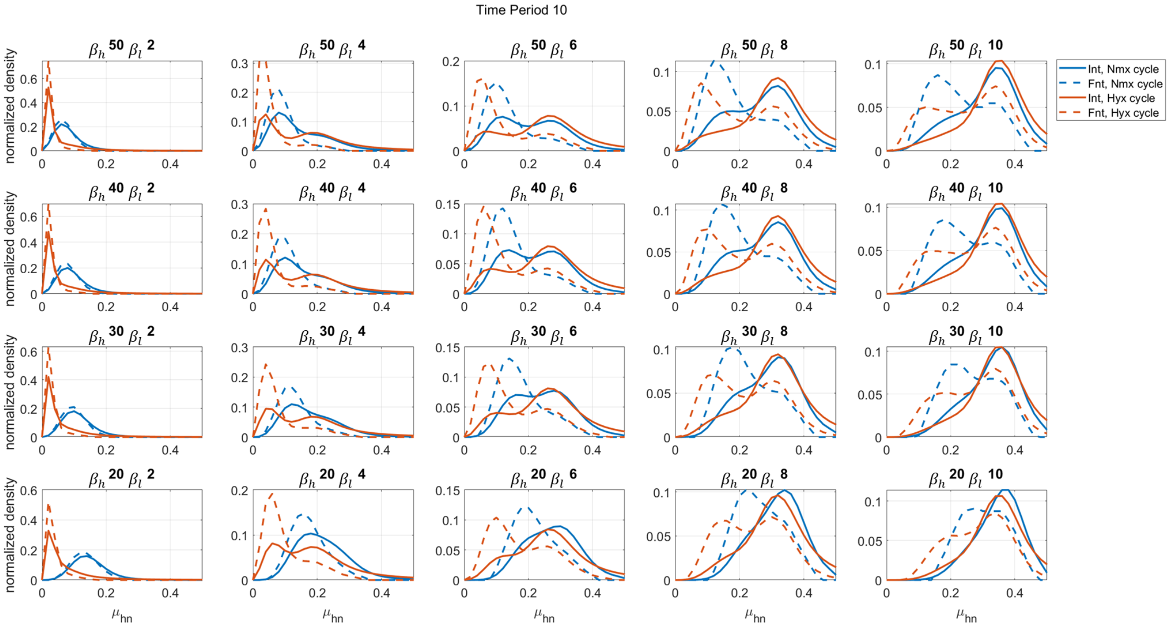


**Fig. S5**: Normalised phenotypic ($\mu_{hn}$) distribution of cells in the bulk (total population) and front (defined by width of 0.5 unit distance from the right boundary) analysed at the end of the normoxic and hypoxic cycles of the last period of the simulation. Here the environment period is 10.

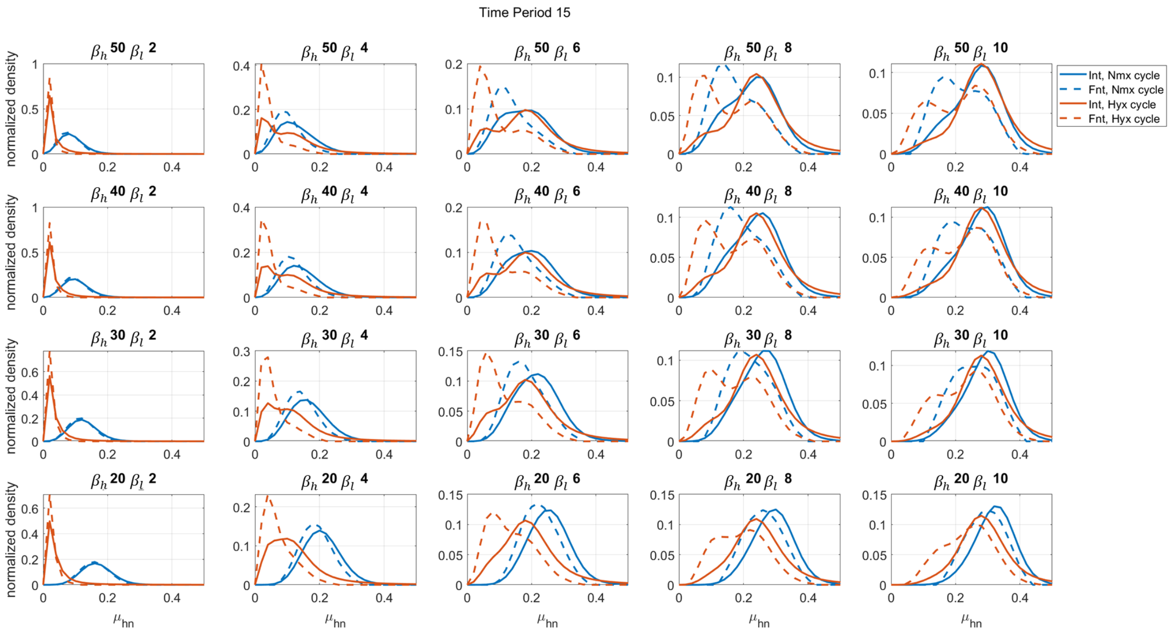


**Fig. S6**: Normalised phenotypic ($\mu_{hn}$) distribution of cells in the bulk (total population) and front (defined by width of 0.5 unit distance from the right boundary) analysed at the end of the normoxic and hypoxic cycles of the last period of the simulation. Here the environment period is 15.

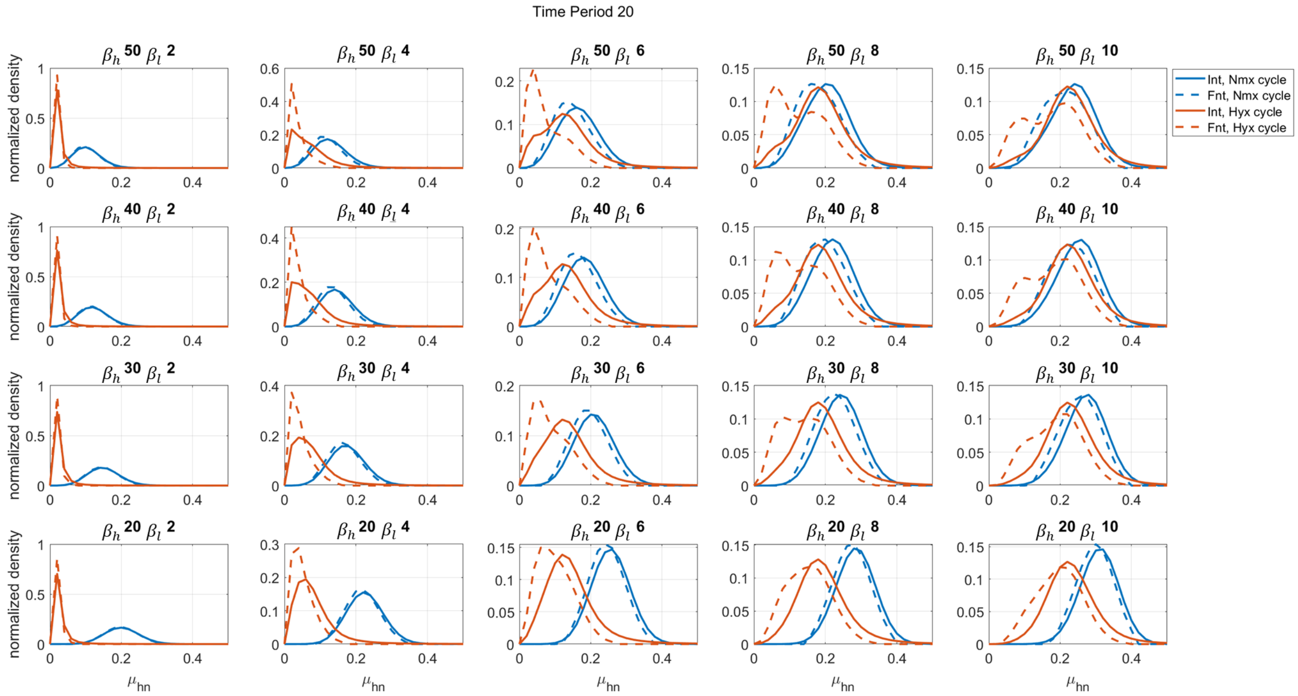


**Fig. S7**: Normalised phenotypic ($\mu_{hn}$) distribution of cells in the bulk (total population) and front (defined by width of 0.5 unit distance from the right boundary) analysed at the end of the normoxic and hypoxic cycles of the last period of the simulation. Here the environment period is 20.

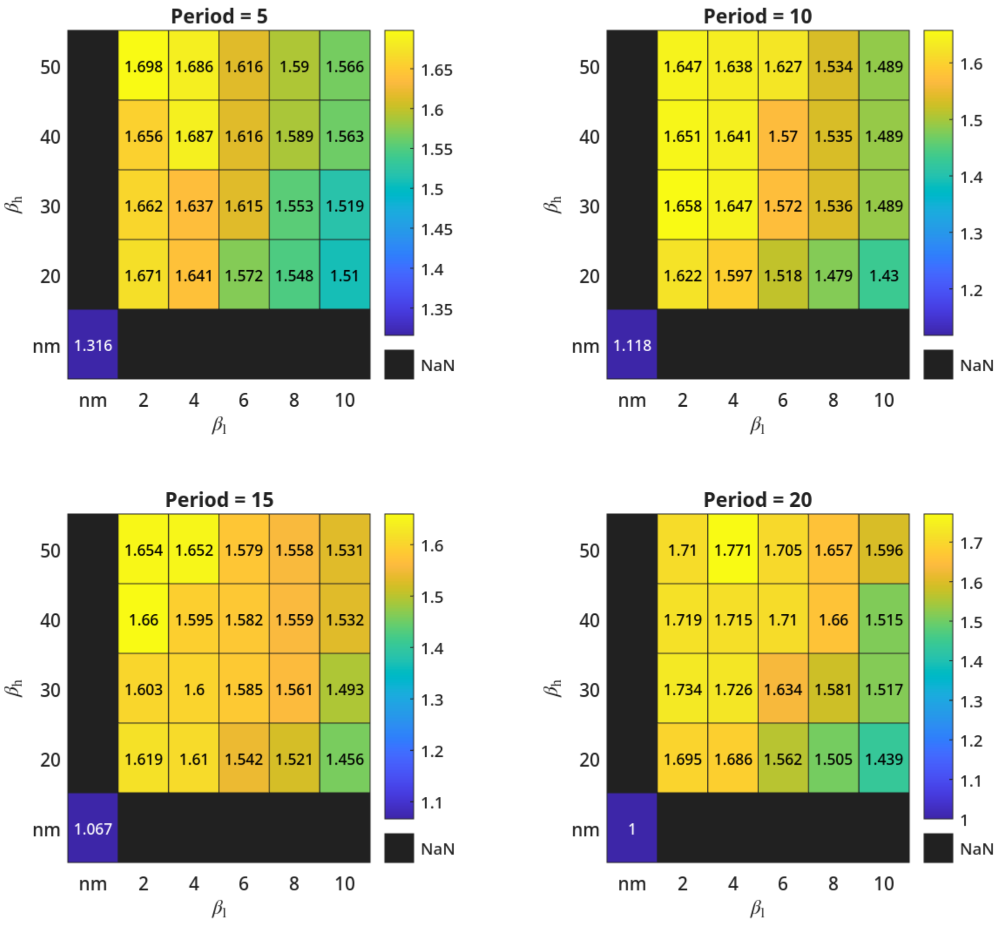


**Fig. S8**: Sum of the normalized tumor volume at $t_{\text{end}} = 60$ and the normalized value of $(t_{\text{end}} - t_{x=9.62})$ as functions of $\beta_h$ and $\beta_l$ for different periods of cyclic hypoxia. Each quantity is normalized by its respective maximum value.
